# Elevated M-MDSCs in Circulation Are Indicative of Poor Prognosis in Diffuse Large B-Cell Lymphoma Patients

**DOI:** 10.3390/jcm10081768

**Published:** 2021-04-19

**Authors:** Zhitao Wang, Rui Jiang, Qian Li, Huiping Wang, Qianshan Tao, Zhimin Zhai

**Affiliations:** 1 Department of Hematology, The Second Affiliated Hospital of Anhui Medical University, Hefei 230601, China; wangzhitao@stu.ahmu.edu.cn (Z.W.); aydxyk101@163.com (Q.L.); ahfymb1989@163.com (H.W.); ahbengbuwyl@163.com (Q.T.); 2 Department of Hematology, The 901st Hospital of the Joint Logistics Support Force of PLA, Hefei 230032, China; jianglang2002@sina.com

**Keywords:** myeloid-derived suppressor cells, diffuse large B-cell lymphoma, interleukin-35, tumor progression, prognosis, immunosuppression

## Abstract

Myeloid-derived suppressor cells (MDSCs) are defined as negative regulators that suppress the immune response through a variety of mechanisms, which usually cluster in cancer, inflammation, and autoimmune diseases. This study aims to investigate the correlation between M-MDSCs and the clinical features of diffuse large B-cell lymphoma (DLBCL) patients, as well as the possible accumulation mechanism of M-MDSCs. The level of M-MDSCs is significantly increased in newly diagnosed and relapsed DLBCL patients. Regarding newly diagnosed DLBCL patients, the frequency of M-MDSCs is positively correlated with tumor progression and negatively correlated with overall survival (OS). More importantly, the level of M-MDSCs can be defined as a biomarker for a poor prognosis in DLBCL patients. Additionally, interleukin-35 (IL-35) mediates the accumulation of M-MDSCs in DLBCL patients. Anti-IL-35 treatment significantly reduces levels of M-MDSCs in Ly8 tumor-bearing mice. Thus, M-MDSCs are involved in the pathological process of DLBCL. Targeting M-MDSCs may be a promising therapeutic strategy for the treatment of DLBCL patients.

## 1. Introduction

Diffuse Large B-cell lymphoma (DLBCL) is the most common subtype of non-Hodgkin lymphoma, which represents about 30–40% of lymphomas [1]. The chemotherapy regimen of CHOP, defined as standard cyclophosphamide, doxorubicin, vincristine, and prednisolone or R-CHOP in combination with rituximab (R), has significantly improved survival outcomes in DLBCL patients [2]. However, approximately 30% of DLBCL patients in stage III and IV remain intractable, and the disease could eventually relapse [3]. During recent decades, the most commonly used standard indicator to assess prognosis in DLBCL is the international prognostic index (IPI), which is based on clinical parameters, including age, Eastern Cooperative Oncology Group(ECOG)performance status, Lactate Dehydrogenase (LDH)level, number of extranodal sites, and Ann Arbor stage. However, final survival is different in patients with identical IPI scores [4]. This means the IPI score system needs further improvement. It is notable that the IPI score system does not include the host tumor microenvironment (TME). The clinical outcome is dependent on many factors, including tumor histologic aggressiveness, immunologic status, and the tumor microenvironment, especially the immunosuppressive regulators [5].

MDSCs is a heterogeneous, immature immunosuppressive cell population which plays an important role in the occurrence and progression of tumors [6,7]. Considering mice, MDSCs can be divided into two types according to the expression of Gr-1, including the subtypes of Ly6G and Ly6C, which can be described as CD11b^+^Ly6C^low^Ly6G^+^ PMN-MDSCs and CD11b^+^Ly6C^high^Ly6G^−^M-MDSCs [8]. No human equivalent of Gr-1 exists, so there is no uniform immunophenotype of human MDSCs. MDSCs promote tumor progression by inhibiting host immune responses [9]. MDSCs exert their immunosuppressive effect through different mechanisms, including direct contact and secreting immunomodulatory factors [10,11]. MDSCs have been described in hematological malignancies, including lymphoma [12], leukemia [13] and multiple myeloma [14]. However, the clinical significance of MDSCs in DLBCL has not been well investigated, especially combined with clinical parameters and prognoses.

It has been proven that MDSCs are found to accumulate in the presence of several biologic factors, such as cytokines, tumor cells, and complement proteins [15]. MDSCs can be generated by interleukin-6 (IL-6) and Granulocyte-macrophage Colony Stimulating Factor (GM-CSF)in vitro [16]. Interleukin-35 (IL-35), composed of an IL-27 subunit EBI3 and an IL-12 subunit p35, is an immunosuppressive cytokine defined as a negative regulator of T cell response [17,18]. Previously, it was reported that IL-35 could induce MDSC accumulation in the tumor microenvironment and promote tumor angiogenesis [19]. However, the role of IL-35 in the pathogenesis of DLBCL, as well as the effect on MDSCs accumulation, have not been well studied.

Here, we investigate the level and possible accumulation mechanism of M-MDSCs, which may be defined as a prognostic biomarker in DLBCL patients.

## 2. Materials and Methods

### 2.1. Patient and Sample

During January 2014 to June 2020, peripheral blood was collected from 65 newly diagnosed, 12 relapsed, 26 remission DLBCL patients, and 30 healthy donors when the state of the disease was defined, as shown in Table 1. All newly diagnosed DLBCL were classified according to World Health Organization standards [20]. Eighteen newly diagnosed DLBCL patients, who received at least 4 cycles of CHOP or R-CHOP regimen and had a complete response, were successfully followed up. Blood samples were obtained before receiving the 5th regimen. The response was assessed according to the RECIL-2017 for lymphoma [21]. Patients with other medical conditions, including infectious diseases, autoimmune diseases, and other types of tumors, were excluded from the study. According to the guidelines of the Helsinki Declaration, the Ethics’ Department of our University committee approved the research program. All patients and volunteers gave written informed consent.

### 2.2. Flow Cytometry (FCM) Analysis

Monoclonal antibodies (mAbs) were purchased from Beckman Coulter–Immunotech: FITC-labeled anti-CD14 (clone No. 116), APC-labeled anti-CD14 (clone No. RMO52), PE-labeled anti-HLA-DR (clone No. B8.12.2), ECD-labeled anti-HLA-DR (clone No. Immu-375), PE-labeled anti-CD4 (clone No. 13B8.2); Mouse FITC-labeled anti-Ly6G (clone No. RB6–8C5), Mouse APC-labeled anti-Ly6C (clone No. ab93550), and Mouse PE-labeled anti-CD11b (clone No. M1/70).

Peripheral blood mononuclear cells (PBMCs) were layered over Ficoll-Hypaque (Amersham Biosciences, Sweden) and centrifuged at 500× *g* for 25 min. Following density gradient centrifugation, PBMCs and plasma were collected. One hundred microliters of PBMCs were used for flow cytometry (Flow Cytometer: FC500 MPL, Beckman Coulter, Brea, CA, USA) and the rest for real-time PCR analysis. Ten thousand cells were analyzed by FCM for each sample. Isotype-matched antibodies were used as controls. During this study, the immunophenotype of M-MDSCs was defined as CD14^+^HLA-DR^−/low^ in humans and CD11b^+^Ly6C^+^Ly6G^−^ in mice.

### 2.3. Cytokine Assay

A human IL-35 enzyme linked immunosorbent assay (ELISA) kit (Biolegend, San Diego, CA, USA) was used to measure the concentration of IL-35 according to the instructions. Each sample was run in duplicate.

### 2.4. RT-PCR Analysis

TRizol reagent (Invitrogen, Carlsbad, CA, USA) was used to extract total RNA from PBMCs. First-strand cDNA was synthesized using the First-Strand Synthesis System (TaKaRa, Dalian, China). SYBR Green PCR Master mix (TaKaRa, Dalian, China) was used to perform the Real-time PCR.

An Applied Biosystems 7500 Real-time Polymerase Chain Reaction (RT-PCR) system was used to analyze the subunit EBI3 and p35 of IL-35. The following primers were employed in each reaction: EBI3, forward 5′-GGCAAGTAGCAAG GGCTTC-3′ and reverse 5′-AGTCGGTCATCTGAGGTTGC-3′; p35, forward 5′-TCCTCCTTGAAGAACCGGA-3′ and reverse 5′-TGA CAACGGTTTGGAGGGAC-3′. Glyceraldehyde phosphate dehydrogenase (GAPDH) was used as the control: forward 5′-CAGGAGGCCATTGCTGATGAT-3′ and reverse 5′-GAAGGCTGGGGCTCATTT-3′. The thermal cycling conditions are described as follows: Following an initial denaturation step at 95 °C for 30 s, 40 cycles of profile were carried out: 95 °C, 5 s; 60 °C, 34 s. Relative transcripts were determined by the formular: 2^−(CTtarget-CTcontrol)^.

### 2.5. Cell Culture and Cytokine Induction

PBMCs (1 × 10^6^) were isolated from 5 healthy controls and incubated in the presence or absence of rh-IL-35 (Recombinant Human Interleukin-35, 50 ng/mL, Sino Biological Inc., Beijing, China) in a 24-well plate for 72 h in vitro. To enhance cell viability, rh-GM-CSF (10 ng/mL; Sigma, St. Louis, MI, USA) was added to the mixture [22]. PBMCs were cultured alone as a control. Three replicates were performed for each condition. The cells were cultured at 37 °C with an RPMI-1640 medium in a humidified CO_2_-containing atmosphere. 

### 2.6. Assay for Autologous T-Cell Proliferation

An MoFlo XDP cell sorter (Beckman Coulter, USA) was used to isolate CD14^+^HLA-DR^−/low^MDSCs(M-MDSCs) and CD14^+^HLA-DR^+^cells. Autologous CD4^+^T cells were sorted by anti-CD4 beads (Miltenyi Biotec, Bergisch Gladbach, Germany) from the same healthy control. The purity of sorted cells was >95%. 

CD4^+^T cells were incubated with CFSE (0.5 μM, Invitrogen, USA). Next, M-MDSCs and CD14^+^HLA-DR^+^cells were cocultured with CFSE-labeled CD4^+^T cells, respectively, in a 96-well plate at the ratio of 1:1. CD4^+^ T cells were cultured alone as a positive control. All cells were cultured in an RPMI-1640 medium with anti-CD3 (2 μg/mL), anti-CD28 (5 μg/mL). The suppressive ability of M-MDSCs on CD4^+^T cells was analyzed 3 days later. Supernatants of culture were obtained and stored at −80 °C until used. Interferon-γ (IFN-γ) was detected using an ELISA Kit (R&D System; ESM, Minneapolis, MN, USA).

### 2.7. Animal Models and Treatments

To establish the Ly8 DLBCL tumor mouse model, 1 × 10^6^ Ly8 cells were subcutaneously injected into the flank of NOD-SCID mice to form tumors. Seven days after tumor cell injection (when the tumor surface area was ~100 mm^2^), mice received weekly administration of anti-IL-35 (Clone V1.4F5.25) or IgG_2b_ antibody (100 μg) where indicated. Additional NOD-SCID mice without any treatment were regarded as the control group. According to the experimental scheme, the mice were killed on Day 23 after tumor inoculation, and blood was collected. Blood was subsequently prepared for flow cytometry analysis of the M-MDSCs population.

### 2.8. Statistical Analysis

Statistical analysis was conducted with the SPSS 17.0 software (SPSS Inc, Chicago, IL, USA). The Mann-Whitney U-test, Student’s t test, and one-way ANOVA were used to determine the statistical significance when appropriate. To evaluate the correlations, Spearman’s coefficient test was used. The overall survival rate (OS) was defined as the time from the start of treatment to death from any cause. The OS was determined using the Kaplan-Meier method. The difference was assessed using the log-rank test. A COX proportional hazard regression analysis identified independent prognostic factors for OS. Regarding all analyses, a *p* value <0.05 was considered significant.

## 3. Results

### 3.1. Increased M-MDSCs in Newly Diagnosed and Relapsed DLBCL Patients

**Figure 1:**
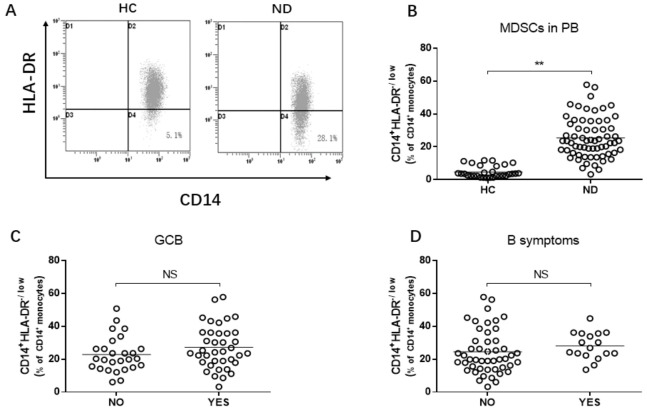
The level of M-MDSCs in DLBCL patients. (**A**) FCM dot plots demonstrate the frequency of M-MDSCs. (**B**) M-MDSCs in DLBCL patients compared to healthy controls. (**C**) M-MDSCs in GCB and no-GCB DLBCL patients. (**D**) M-MDSCs in A and B symptoms in DLBCL patients. HC, healthy control; ND, newly diagnosed. ** *p* < 0.01.

Cells with the immunophenotype of CD14^+^HLA-DR^−/low^ were defined as monocytic MDSCs (M-MDSCs). Compared to the 30 healthy controls, a significantly increased frequency of M-MDSCs was found in the 65 newly diagnosed DLBCL patients (4.7 ± 3.6% vs. 25.4 ± 12.3%, *p* < 0.01, Figure 1A,B). However, no significant difference existed between the groups by age, gender, and GCB, or B symptoms (Figure 1C,D).

### 3.2. M-MDSCs Levels Correlate with Disease Progression in Newly Diagnosed DLBCL Patients

**Figure 2:**
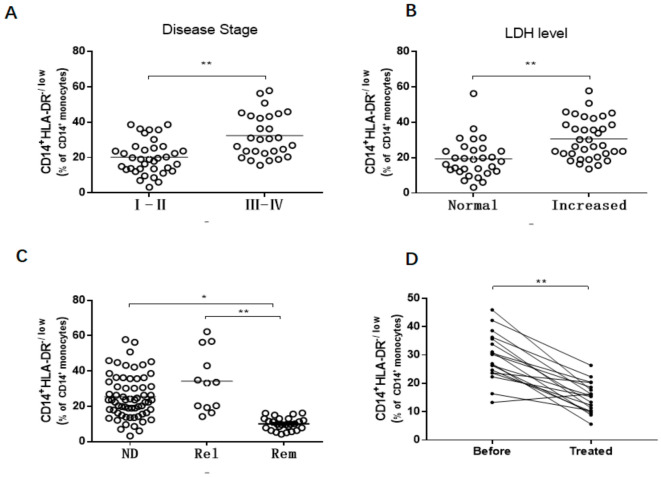
M-MDSCs were associated with tumor progression in DLBCL patients. (**A**) M-MDSCs were associated with DLBCL clinical stages. (**B**) High LDH level DLBCL patients had an increased frequency of M-MDSCs. (**C**) Newly diagnosed and relapsed patients had higher levels of M-MDSCs than remission patients. (**D**) The levels of M-MDSCs significantly decreased after therapy. ND, newly diagnosed; Rel, relapsed; Rem, remission. * *p* < 0.05; ** *p* < 0.01.

Many clinicopathological factors were used to indicate disease progression, including the Ann Arbor Stage, LDH level, and disease status. DLBCL patients with stage III–IV were found to have higher levels of M-MDSCs compared to patients with early stage I–II (32.4 ± 12.2% vs. 20.1 ± 9.5%, *p* < 0.01, Figure 2A). Higher levels of M-MDSCs were observed in DLBCL patients with increased LDH levels (30.6 ± 11.3%) compared with the LDH normal group (19.4 ± 10.7%, *p* < 0.01, Figure 2B). An increased frequency of M-MDSCs existed in newly diagnosed (25.4 ± 12.3%) and relapsed (34.2 ± 17.0%) DLBCL patients compared to remission (10.1 ± 3.5%, Figure 2C) patients. Regarding the 18 patients who received four cycles of CHOP or R-CHOP regimens of chemotherapy, the frequency of M-MDSCs was significantly decreased after therapy (28.6 ± 8.5% vs. 13.2 ± 5.3; *p* < 0.01, Figure 2D). 

### 3.3. The Association Between M-MDSCs and Prognosis of DLBCL Patient

**Figure 3:**
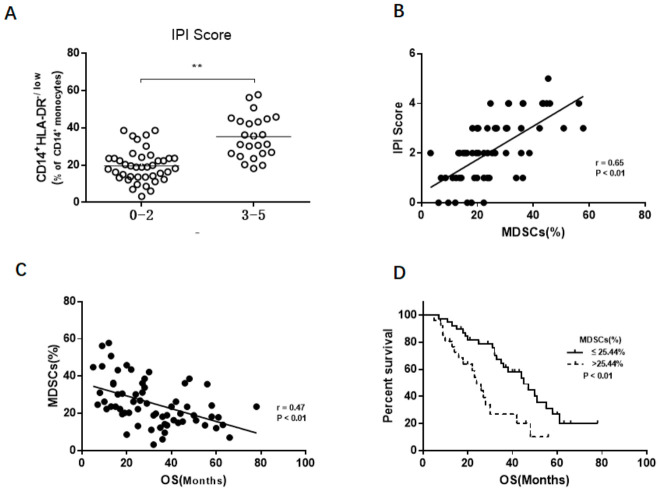
Circulating M-MDSCs was a prognostic factor in DLBCL patients. (**A**) High IPI score DLBCL patients had an increased frequency of M-MDSCs. (**B**) M-MDSCs levels were positively associated with the IPI score. (**C**) M-MDSC levels were negatively correlated with the OS. (**D**) Short OS were shown in high M-MDSCs groups. The cut-off value is the median of M-MDSC levels. ** *p* < 0.01.

First, the relationship between the IPI score and the expansion of M-MDSCs was investigated. We found increased levels of M-MDSCs in the high IPI score (IPI score: 3–5) DLBCL patients compared to the low IPI score (IPI score: 0–2) patients (35.3 ± 11.4% vs. 19.6 ± 8.7; *p* < 0.01, Figure 3A). Furthermore, the IPI score was significantly and positively associated with the frequency of M-MDSCs (r = 0.65, *p* < 0.01, Figure 3B).

The follow-up time was 5–76 months from January 2014 to June 2020. The negative correlation between the OS and the frequency of M-MDSCs was further validated (r = 0.47, *p* < 0.01, Figure 3C). Based on the median value frequency of M-MDSCs (25.4%), DLBCL patients were divided into two groups. Concerning the low group (*n* = 39), M-MDSCs levels were defined as less than or equal to 25.4%. Regarding the high group (*n* = 26), M-MDSCs levels were greater than 25.4%. A Kaplan-Meier analysis showed the OS of DLBCL patients with low M-MDSC levels was significantly longer than those with high M-MDSC levels (*p* < 0.01, Figure 3D). 

A univariate analysis of prognostic factors for OS, including age, gender, disease stage, LDH level, B symptoms, GCB, IPI score, and the frequency of M-MDSCs, was conducted. Logically, disease stage, IPI score, LDH level, and the frequency of M-MDSCs were significant prognostic indicators for OS (*p* < 0.05; Table 2). When adjusted for the key clinical prognostic factors, a multivariate Cox regression analysis also was performed. The results showed the IPI score and M-MDSC levels were associated with prognosis and could be recognized as independent prognostic factors for DLBCL patients (*p* < 0.05, Table 2).

### 3.4. Increased IL-35 Induce the M-MDSCs Expansion

**Figure 4:**
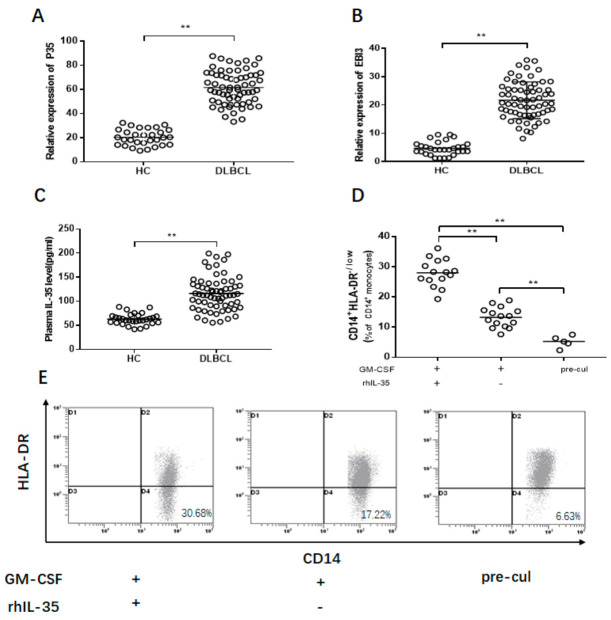
The levels of IL-35 in DLBCL patients and their effect on M-MDSCs. (**A**) p35 and (**B**) EBI3 mRNA expression in DLBCL patients and healthy controls. (**C**) The concentration of IL-35 in DLBCL patients and healthy controls. (**D**) IL-35 induced the expansion of M-MDSCs in vitro. (**E**) FACS dot plots show the proliferation of M-MDSCs induced with IL-35. Pre-cul, pre-culture; ** *p* < 0.01.

IL-35, as a novel inhibitory cytokine, is composed of the subsets of p35 and EBI3 [23]. First, relative expressions of p35 and EBI3 mRNA were detected. Significantly increased expression of p35 mRNA was found in DLBCL patients compared to the healthy controls (61.49 ± 13.58 vs. 20.34 ± 7.06; *p* < 0.01, Figure 4A), as well as expression of EBI3 mRNA (21.88 ± 6.06 vs. 4.76 ± 2.43; *p* < 0.01, Figure 4B). Next, the concentration of IL-35 was investigated. There was a significantly higher concentration of IL-35 in DLBCL patients compared to the healthy controls (103.39 ± 57.53 pg/mL vs. 65.18 ± 18.23 pg/mL; *p* < 0.01, Figure 4C).

Next, the effect of IL-35 on the accumulation of M-MDSCs was investigated. First, PBMCs, isolated from healthy controls, were cultured with rhIL-35(50 ng/mL) for 72 h in vitro. GM-CSF (10 ng/mL; Sigma) was used to support cell viability. The percentage of M-MDSCs was significantly increased with stimulation of IL-35 compared to the control (*p* < 0.01, Figure 4D,E).

### 3.5. IL-35-Induced M-MDSCs Suppress CD4^+^ T Cell Response

**Figure 5:**
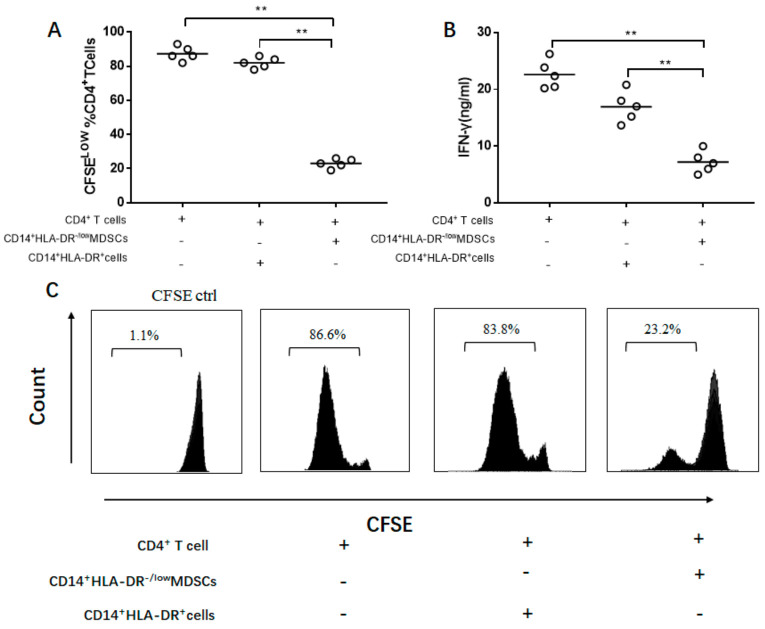
Functional analysis of M-MDSCs on CD4^+^T cells. (**A**) CFSE-labeled CD4^+^T cells were suppressed by M-MDSCs, (**B**) associated with a reduction of IFN-γ. (**C**) A flow cytometry histogram shows the suppressive activity of M-MDSCs. Ctrl, control, ** *p* < 0.01.

Compared with the CD14^+^HLA-DR^+^ cells, M-MDSCs had a strong ability to suppress CD4^+^T cell proliferation and reduce the production of IFN-γ (*p* < 0.01, Figure 5A,B). CD14^+^HLA-DR^+^cells did not have this capacity (*p* < 0.01, Figure 5C).

### 3.6. Anti-IL-35 Treatment Block M-MDSC Expansion In Vivo

**Figure 6:**
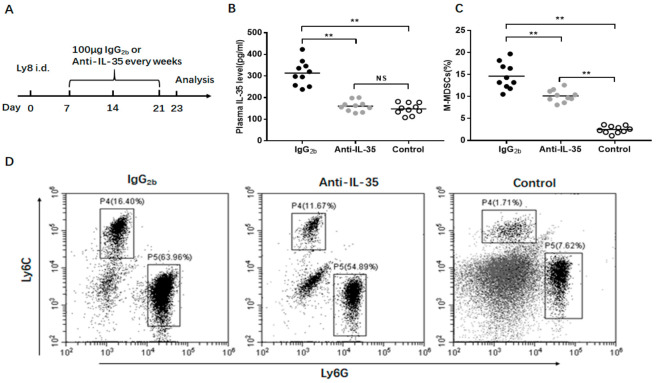
Effect of IL-35 on M-MDSC expansion in vivo. (**A**) Schematic representation of the experimental design. (**B**) Anti-IL-35 treatment reduced levels of IL-35. (**C**) Anti-IL-35 blocked the M-MDSC expansion in vivo. (**D**) Flow cytometry dot plots show the level of M-MDSCs in the three groups (P4 region). ** *p* < 0.01.

To study the effect of IL-35 on MDSC expansion, NOD-SCID mice injected with Ly8 DLBCL tumor cells were treated with anti-IL-35 or IgG_2b_ antibodies (Figure 6A). Additional NOD-SCID mice without any treatment were regarded as the control group. Compared with the IgG2b antibody, Anti-IL-35 treatment significantly reduced the plasma levels of IL-35 in Ly8 tumor-bearing mice (*p* < 0.01, Figure 6B). Furthermore, obviously decreased levels of M-MDSCs were found in mice treated with Anti-IL-35 (*p* < 0.01, Figure 6C,D, P4 region).

## 4. Discussion

MDSCs are a heterogeneous, immature immunosuppressive cell population, which has a positive role in tumorigenesis and tumor progression [24,25]. It has been proven that the frequency of MDSCs in colorectal cancer [26], breast cancer [27], and multiple myeloma [14] is significantly increased and closely related to the progression of the tumor. The immunosuppressive capacity of M-MDSCs was more intensely compared with G-MDSCs in the tumor microenvironment [28]. Additionally, G-MDSCs were absent from PBMCs, and were only detected in whole blood in several malignancy patients [29]. Thus, G-MDSCs are less studied than M-MDSCs in previous reports. M-MDSCs focused our attention during this study. 

During this study, the clinical parameters and prognostic significance associated with the frequency of M-MDSCs was investigated in patients with DLBCL. First, the frequency of M-MDSCs was significantly increased in newly diagnosed and relapsed DLBCL patients and closely related to disease progression (disease stage, LDH levels, and IPI score). Following chemotherapy, a significant decrease in the levels of M-MDSCs was found. This indicated that the level of M-MDSCs reflected the disease progress of DLBCL patients. Accompanying the reduction of the tumor burden, inflammatory cytokines secreted by DLBCL cells significantly decreased, accompanied by reduced levels of M-MDSCs. Concerning DLBCL patients, significant changes in M-MDSCs can be used to indicate the outcome of chemotherapy.

During a previous study [30,31], the levels of MDSCs were significantly different between GCB and non-GCB in poor and very good risk groups in DLBCL patients. Following chemotherapy, no significant difference existed in the five-year OS between GCB and non-GCB-DLBCL patients. The levels of M-MDSCs can be indicated as a biomarker to evaluate the prognosis of DLBCL patients. During the present study, the frequency of M-MDSCs was positively associated with the IPI score and negatively correlated with the OS in DLBCL patients. The group with a longer OS had a lower frequency of M-MDSCs. This proved that the level of M-MDSCs was a factor affecting the OS in DLBCL patients. The results from a Cox regression analysis showed that M-MDSCs were associated with a poor prognosis and could be defined as a prognostic biomarker for DLBCL patients.

Previous studies have shown that IL-35 exerts an immunosuppressive effect on inflammation related diseases [18]. The concentration of IL-35 increased in various tumor patients, including colorectal cancer [32], acute myeloid leukemia [17], and hepatocellular carcinoma [33]. However, to our best knowledge, there were few reports investigating IL-35 in DLBCL patients, as well as the effect of IL-35 on MDSCs. Wang et al. found that IL-35 has a strong ability to induce CD11b^+^Gr1^+^ myeloid cell accumulation in the mouse tumor microenvironment [19]. The influence of IL-35 on the accumulation of human M-MDSCs has not been reported before, to our knowledge. Data from the present study demonstrated that the concentrations of IL-35, as well as p35 and EBI3 mRNA expressions, were significantly elevated in DLBCL patients. Additionally, IL-35 has an ability to induce M-MDSC accumulation. Regarding the experiment in mice, the direct depletion of IL-35 using a neutralizing antibody contributes to the decrease in M-MDSC accumulations in mice with Ly8 DLBCL tumors. Interestingly, a decreased level of G-MDSCs also was found with the treatment of Anti-IL-35. These results collectively demonstrated that Anti-IL-35 treatment significantly blocked M-MDSC expansion. IL-35 exerted an immunosuppressive effect by inducing M-MDSC accumulations. Recently, however, the specific mechanism and molecular pathway of IL-35-induced M-MDSC expression remains to be further elucidated.

The uniform phenotypic marker for MDSCs is still lacking. The critical feature in the identification of MDSCs was the immunosuppressive capacity [34]. To identify whether the IL-35 induced CD14^+^HLA-DR^−/low^ cells have immunosuppressive effect, it was necessary to evaluate the immunosuppressive activities of CD14^+^HLA-DR^−/low^ cells on autologous T cell proliferation and IFN-γ production. It was found that CD14^+^HLA-DR^−/low^ cells significantly suppressed the proliferation of CD4^+^ T cells compared to the CD14^+^HLA-DR^+^ cells. Moreover, the production of IFN-γ was significantly decreased in CD4^+^ T cells co-cultured with CD14^+^HLA-DR^−/low^ cells. Strikingly, CD14^+^HLA-DR^−/low^ cells exerted a strong suppressive ability on T cell proliferation and IFN-γ production. Hence, CD14^+^HLA-DR^−/low^ cells could be considered as M-MDSCs in DLBCL patients.

## 5. Conclusions

Increased levels of M-MDSCs are positively associated with tumor progression and inversely correlated with OS in DLBCL patients. The level of M-MDSCs is associated with prognosis and could be defined as a prognostic indicator for DLBCL patients. Additionally, IL-35 has an obvious ability to induce the accumulation of M-MDSCs. Therefore, M-MDSCs participate in immune escape and have a prognostic value in DLBCL patients. Targeting M-MDSCs may be a promising therapeutic strategy for DLBCL patients.

## Figures and Tables

**Table 1:** Characteristics of healthy donors and DLBCL patients.

State of Disease at Sample Draw	No. of Patients	Average Age (Range)	Gender(M/F)
Newly diagnosed	65	58.7(28–80)	40/25
Disease stage			
I-II	37	57.8(30–80)	20/17
III-IV	28	59.1(28–78)	20/8
B symptoms			
YES	17	52.3(28–69)	9/8
NO	48	61.4(32–80)	31/17
GCB			
YES	38	58.9(28–76)	23/15
NO	27	58.5(30–80)	17/10
LDH			
Normal	30	60.4(30–80)	20/10
Increased	35	57.3(28–78)	20/15
IPI score			
0–2	41	55.8(28–76)	24/17
3–5	24	63.7(32–80)	16/8
Relapsed	12	58.3(36–62)	9/3
Remission	26	62.3(31–76)	14/12
Healthy donors	30	60.1(30–78)	18/12

GCB germinal center B-cell-like, LDH lactate dehydrogenase, IPI International Prognostic Index. B symptoms: B symptoms refer to systemic symptoms of fever, night sweats, and weight loss which can be associated with diffuse Large B-cell lymphoma (DLBCL).

**Table 2:** Univariate and Multivariate analysis of prognostic factors in patients with DLBCL.

Factor		Univariate Analysis			Multivariate Analysis	
	HR	95% CI	*p*	HR	95% CI	*p*
Age (year)						
(≤60 vs. >60)	1.511	0.811–2.817	0.194			
Gender						
(Male vs. Female)	1.127	0.601–2.112	0.709			
Disease stage						
(I-II vs. III-IV)	3.026	1.591–5.755	0.001	2.184	0.916–5.205	0.078
B symptoms						
(No vs. Yes)	1.339	0.710–2.523	0.367			
GCB						
(No vs. Yes)	1.060	0.576–1.981	0.855			
LDH						
(Normal vs. Increased)	0.348	0.136–0.887	0.027	1.461	0.529–4.035	0.464
IPI score						
(0–2 vs. 3–5)	2.976	1.549–5.717	0.001	0.271	0.103–0.712	0.008
MDSCs						
(≤25.44% vs. >25.44%)	2.707	1.409–5.199	0.003	2.682	1.198–6.004	0.016

OS overall survival, HR hazard ratio, CI confidence interval, GCB germinal center B-cell-like, LDH lactate dehydrogenase, IPI International Prognostic Index. MDSC level (high/low) is based on the median value of the MDSC frequency.

## Data Availability

The data presented in this study are available on request from the corresponding author.
